# A comparison and optimization of methods and factors affecting the transformation of *Escherichia coli*

**DOI:** 10.1042/BSR20130098

**Published:** 2013-12-12

**Authors:** Weng-Tat Chan, Chandra S. Verma, David P. Lane, Samuel Ken-En Gan

**Affiliations:** *Bioinformatics Institute, Agency for Science, Technology, and Research (A*STAR), Singapore 138671; †Department of Biological Sciences, National University of Singapore (NUS), Singapore 119077; ‡School of Biological Sciences, Nanyang Technological University (NTU), Singapore 639798; §p53 Laboratory, Agency for Science, Technology, and Research (A*STAR), Singapore 138648

**Keywords:** CaCl_2_ method, competent bacteria, DMSO method, Hanahan's method, heat shock, CaCl_2_, calcium chloride, Cfu, colony-forming units, *E. coli*, *Escherichia coli*, FSB, frozen storage buffer, LB, Luria–Bertani (broth), PEG, polyethylene glycol, SOB, super optimal broth, SOC, super optimal broth with catabolite repression, TrE, transformation efficiency, TSB, transformation storage buffer

## Abstract

DNA manipulation routinely requires competent bacteria that can be made using one of numerous methods. To determine the best methods, we compared four commonly used chemical methods (DMSO, MgCl_2_–CaCl_2_, CaCl_2_ and Hanahan's methods) on frequently used *Escherichia coli* (*E. coli*) strains: DH5α, XL-1 Blue, SCS110, JM109, TOP10 and BL21-(DE3)-PLysS. Hanahan's method was found to be most effective for DH5α, XL-1 Blue and JM109 strains (*P*<0.05), whilst the CaCl_2_ method was best for SCS110, TOP10 and BL21 strains (*P*<0.05). The use of SOB (super optimal broth) over LB [Luria–Bertani (broth)] growth media was found to enhance the competency of XL-1 Blue (*P*<0.05), dampened JM109′s competency (*P*<0.05), and had no effect on the other strains (*P*>0.05). We found no significant differences between using 45 or 90 s heat shock across all the six strains (*P*>0.05). Through further optimization by means of concentrating the aliquots, we were able to get further increases in transformation efficiencies. Based on the optimized parameters and methods, these common laboratory *E. coli* strains attained high levels of TrE (transformation efficiency), thus facilitating the production of highly efficient and cost-effective competent bacteria.

## Abstract 2

This research paper aims to investigate the factors affecting the production of competent cells to produce high levels of transformation efficiency in common laboratory strains.

## INTRODUCTION

Bacterial transformation, the process whereby bacteria are able to take up foreign DNA was first demonstrated by Griffith for *Streptococcus pneumonia* [1], and is now routinely used in laboratories. Although reported to occur naturally in bacteria such as *Bacillus subtilis* [2], such phenomenon is generally uncommon in *Escherichia coli*, which require induction by artificial methods, such as those first demonstrated by Mandel and Higa [3]. This chemical method involved treating the bacteria with bacteriophage *λ* DNA in the presence of Ca^2+^ ions, followed by a brief heat shock [4]. This was referred to as the ‘calcium chloride’ (CaCl_2_) method [5] and was subsequently adopted to impart antibiotic resistance into the competent *E. coli*. However, it was discovered that only 1–2% of the transformants survived in this method [6], making it inefficient for routine DNA manipulation. Later, modifications such as the use of multiple heat-shock cycles [7] and variations of chemicals were tested. These resulted in several optimized versions of the original CaCl_2_ method [8,9] that typically yield ‘TrE’ (transformation efficiencies) of 5×10^6^–2×10^7^ colonies forming units per microgram (cfu/μg) of DNA.

In 1983, Douglas Hanahan proposed a new method that yielded competency levels of 1–5×10^8^ cfu/μg across many *E. coli* strains [10,11]. However, his method was quite complex, combining several parameters such as the nature of chemicals, plasmids and labware. Subsequently, a simpler method based on the use of DMSO and PEG (polyethylene glycol) (replacing Ca^2+^ ions) and a short incubation of both bacteria and DNA on ice (replacing the heat-shock) was proposed. This method, commonly known as the DMSO method [12], produced up to 1×10^7^–10^8^ cfu/μg. However, to date there has not yet been a comprehensive study comparing the different methods across various strains of *E. coli* to determine the optimal conditions for maximizing the competency of the bacteria. Comparisons are complicated by issues such as the lack of consistency in the methods used to calculate TrE.

In order to address these issues, we examined the following four methods of producing competent bacteria: Hanahan's method [10], the DMSO method [12] and two variants of the CaCl_2_ method [4,13]. Care was taken to ensure that conditions including aliquot volumes, TrE calculations, growth media, heat-shock duration, final resuspension volumes and plasmid were standardized for comparisons. These resulted in establishing the best method for each strain (we tested six different strains of *E. coli*), followed by optimization of other parameters such as growth media and heat-shock incubation times to produce bacteria with TrE levels that are statistically comparable with those reported commercially. To our knowledge, this is the first report of such a comprehensive study since that of Hanahan's (1983 and 1991).

## EXPERIMENTAL

### *E. coli* starting cultures:

Colonies of *E. coli* strains (DH5α – Invitrogen, Cat no. 12297-016, Xl-1 Blue – Stratagene, Cat no. 200247, SCS110 – Stratagene, Cat no. 200249, JM109 – Promega, Cat no. L2001, TOP10 – Invitrogen Cat no. C4040-10, BL21-(DE3)-PLysS – Invitrogen, Cat no. C6060-03) were isolated from commercial sources through plating on LB [Luria–Bertani (broth)] agar (Biopolis Shared Facilities, A*STAR). Single colonies were inoculated into 2 ml of relevant media overnight. Approximately, 100 μl of the cultures were inoculated into 50 ml of pre-warmed respective media to allow growth to early log phase [OD_600_ (optical density) reading of 0.3–0.5) [8,9] (Supplementary Table S1at http://www.bioscirep.org/bsr/033/bsr033e086add.htm), followed by immediate placement on ice for 15 min and centrifugation. The pellets were resuspended in method-specific buffers (see CaCl_2_ [4,13], DMSO [12] and Hanahan's method [10] or supplementary data). All bacteria were grown at 37°C with vigorous shaking at 200–220 rpm (Certomat BS-1, Satorius Stedim Biotech). All centrifugation steps were performed at 3200 ***g*** using Eppendorf, Model 5804R at 4°C.

### Comparison of the four methods on six *E. coli* strains

For comparisons, the following parameters were standardized: (1) Use of 100 μl bacterial aliquots for transformation and TrE calculation; (2) Use of PUC18 plasmid only; (3) Use of LB media for growth and post heat-shock growth; (4) Use of 45 s heat-shock for transformation; and (5) Final resuspension volume of 4 ml from initial 50 ml of bacteria cultures.

### MgCl_2_–CaCl_2_ method: adapted from the method of Sambrook and Russell [13]

Pelleted bacteria from 50 ml cultures were resuspended with gentle pipetting in 15 ml of 0.1 M MgCl_2_ (formulated in de-ionized water and autoclaved) (BDH, VWR International) and incubated on ice for 10 min. The bacteria were pelleted at 4k rpm at 4°C for 10 min, resuspended in 15 ml of 0.1 M CaCl_2_, and incubated on ice for 30 min. After spinning down, the supernatant were discarded, and the pellets gently resuspended in 4 ml (standardized volume) of 0.1 M CaCl_2_ with 20% (v/v) glycerol solution and stored in 100 μl aliquots at −80°C.

### CaCl_2_ method: adapted and modified from the method of Mandel and Higa [4]

Original protocols are in Supplementary Data; at http://www.bioscirep.org/bsr/033/bsr033e086add.htm.

Pelleted bacterial strains from 50 ml cultures were resuspended with gentle pipetting in 25 ml (half the volume of the initial culture) of ice-cold 0.1 M CaCl_2_ (BDH) (formulated in de-ionized water and autoclaved) and incubated on ice for 1 h. The bacteria suspensions were pelleted at 4k rpm at 4°C for 10 min followed by gentle resuspension in 4 ml of 0.1 M CaCl_2_+15% (v/v) glycerol and stored in 100 μl aliquots at −80°C.

### DMSO method: adapted and modified from the metod of Chung and Miller [12]

Original protocols are in Supplementary Data; at http://www.bioscirep.org/bsr/033/bsr033e086add.htm.

Pelleted bacteria strains from 50 ml cultures were resuspended in 4 ml of ice-cold TSB (transformation storage buffer: LB broth at pH 6.1, 10% (w/v) PEG4450, 5% (v/v) DMSO, 10 mM MgCl_2_ and 10 mM MgSO_4_, filter sterilized with 0.45 μm filter) and incubated on ice for 30 min. The bacteria were then subsequently stored in 100 μl aliquots at −80°C.

### Hanahan's method: adapted and modified from Hanahan [10]

Detailed original protocols and FSB (frozen storage buffer) preparation are in Supplementary Data at http://www.bioscirep.org/bsr/033/bsr033e086add.htm.

Pelleted bacterial strains from 50 ml culture were resuspended gently in 16.5 ml of FSB (10 mM CH_3_CO_2_K at pH 7.5, 45 mM MnCl_2_, 10 mM CaCl_2_, 0.1 M KCl, 3 mM [Co(NH_3_)_6_]Cl_3_, 10% glycerol) and incubated on ice for 15 min. The bacteria were then pelleted at 4k rpm at 4°C for 10 min, and resuspended in 4 ml of FSB. 140 μl of DMSO was added twice in intervals of 5 min to the centre of the suspension with gentle swirling. The bacteria suspensions were stored in 200 μl aliquots at −80°C.

### Transformation protocol for the standardized methods

#### 45 s heat-shock transformation – (modified from Stratagene's recommended protocol) – used for all methods except the DMSO method

100 μl of competent bacteria were mixed with 1 μl of control pUC18 plasmid [0.1 ng/μl in nuclease-free water (Agilent, 200231-42)] in cold 14 ml round bottomed tubes (BD, Product no. 352059) and incubated on ice for 30 min. A 42°C heat-shock of 45 s was performed, followed by immediate placement on ice for 2 min. 100 μl of LB media were added to the bacterial suspensions before incubations at 37°C for 1 h. The entire aliquot was plated out on 1.5% (w/v) LB agar plates with 100 μg/ml ampicillin (Goldbio, A-301-5) at 37°C overnight.

#### Transformation of competent bacterial (DMSO method):

100 μl of thawed DMSO competent cells were transferred to ice-cold 14 ml round bottomed tubes (BD, Product no. 352059) and incubated with 0.1 ng/ul of control pUC18 plasmid (Agilent, 200231-42) on ice for 30 min. The cell suspensions were then allowed to grow in 0.9 ml of TSB with 20 mM of glucose at 37°C in vigorous shaking (speed 200–220) for 1 h. The cells were then plated on LB agar plates with 100 μg/ml ampicillin and incubated overnight at 37°C.

### Comparison of the culture media:

#### Optimization of culture media used for starter cultures

The various strains of *E. coli* were induced to be competent using the established best methods as described above, and varying the use of SOB (super optimal broth) [10] or LB [14] as the starting culture media. Heat shock was standardized to 45 s.

### Comparison of the heat-shock incubation times for transformation

The incubation times of the heat shock for all the six strains were made using the exact transformation protocol as described by Hanahan [10] (Supplementary data at http://www.bioscirep.org/bsr/033/bsr033e086add.htm), with the exception of varying the incubation to either 45 or 90 s in the 42°C heat bath.

### Data collection and computation of TrE

All bacterial colonies on the plated agar were counted manually. The TrE were calculated according to the formula provided by Stratagene, and adjusted to aliquot volumes 100 μl.

### Fourfold concentration of optimally induced bacteria

The bacterial strains were concentrated prior to freezing and storage by virtue of a four-fold reduction in the volume of the final storage buffer used to resuspend the strains as per their optimized protocols.

### Statistical computation and analysis

For comparison of the four methods on the six strains, both ANOVA and independent *t* tests were used. ANOVA test was performed to determine the reproducibility of the TrE within each method (Supplementary Table S2A at http://www.bioscirep.org/bsr/033/bsr033e086add.htm), as well as the differences between the four methods (Supplementary Table S2B at http://www.bioscirep.org/bsr/033/bsr033e086add.htm). Independent *t* test was used for the comparison of pair-wise method comparisons (Supplementary Table S2C at http://www.bioscirep.org/bsr/033/bsr033e086add.htm), media (Figure 2), heat-shock incubation times (Figure 3), and four-fold concentration and neat (Figure 4). All statistical analysis was performed using SPSS ver. 17 (IBM) at a 95% confidence interval.

## RESULTS AND DISCUSSION

### Comparison of four different chemical methods of producing competent bacterial strains

TrE for the four different chemical methods mentioned above were tested using 100 μl aliquots, LB growth media, 4 ml final resuspension volumes and 45 s heat-shock duration (with the exception of the DMSO method which uses 10–15 min ice incubation). All the methods yielded TrE that were significantly different from each other (*P*<0.05) (see ANOVA results and independent *t* test results in Supplementary Tables S2B and S2C, respectively) with the exception of the DH5α strain. To test for reproducibility within each method, ANOVA tests (see Supplementary Table S2A) were also performed. The results showed that all strains, again with the exception of DH5α strain (produced using DMSO method; *F*=19.331, *P*= 0.004, Supplementary Table S2A), were reproducible (*P*>0.05).

**Figure 1:**
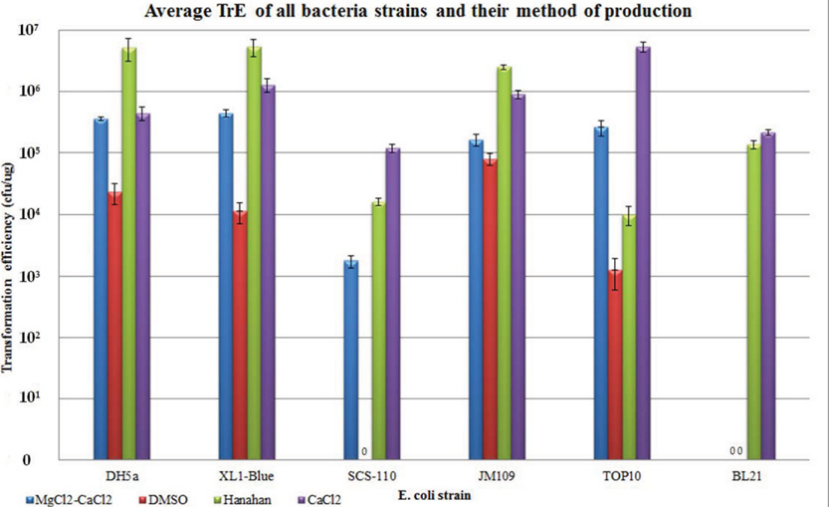
Comparison of the four standardized chemical methods on six strains of *E. coli* Bar chart showing the means and standard errors of the TrE obtained from the six strains of *E. coli* produced using the four different methods. Controls were performed by transforming competent bacteria with water, which did not yield any transformants.

We found that the standardized Hanahan's and CaCl_2_ methods consistently produced higher TrE than the MgCl_2_–CaCl_2_ or the DMSO methods across all the six strains of *E. coli* tested (Figure 1 and Supplementary Table S2). When transformed with pUC18 plasmid, Hanahan's method was most effective for DH5α, XL-1 Blue and JM109 strains, while the CaCl_2_ method was most effective for SCS110, TOP10 and BL21 strains (Figure 1). Surprisingly, repeated attempts (see Supplementary Table S1 for batches made) were still unsuccessful in reproducing the 1×10^7^–10^8^ cfu/μg TrE reported by Chung and Miller [15] for the DMSO method. In fact, neither the SCS110 nor the BL21 strains yielded significant TrE levels for either the DMSO or the MgCl_2_–CaCl_2_ methods (Figure 1). The DH5α strain, produced with the DMSO method, failed to reach TrE levels (10^5^ cfu/μg) suitable for sub-cloning, but did produce better yields (>10^5^ cfu/μg) with both the CaCl_2_ methods, and these yields were statistically similar within the two variant methods (*P*= 0.276).

In agreement with reports suggesting genetic associations with sensitivity to the different methods of chemical induction [11,16], our analysis suggested that modifications of the *gal* operon were associated with the strains that responded better to the CaCl_2_ method. In contrast, modifications of the *relA1* and *gyrA96* genes were associated with increased sensitivity to Hanahan's method (Supplementary Table S3 at http://www.bioscirep.org/bsr/033/bsr033e086add.htm). However, establishment of a firm correlation between the genotype and sensitivity to the chemical induction methods would require more detailed studies.

### Comparison of the use of LB and SOB media and their effects on TrE

**Figure 2:**
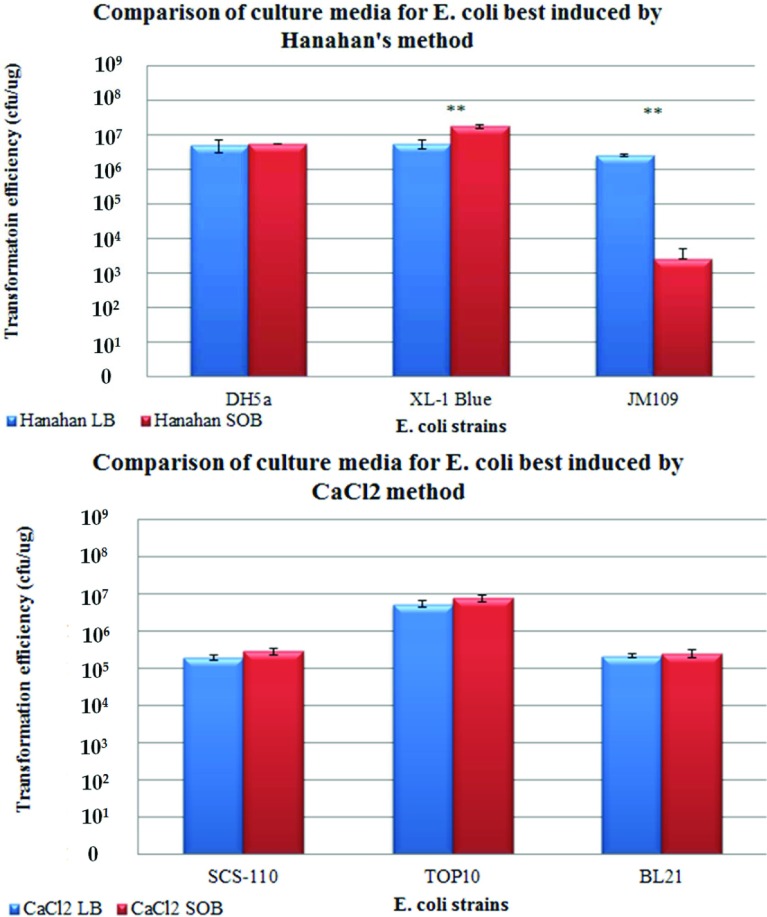
Comparison of the use of LB or SOB as growth media Bar chart showing the means, standard errors and *t* tests of TrE obtained from both CaCl_2_ and Hanahan methods of chemical induction for the respective strains. (Top panel) Bar chart representing the three strains that responded best to Hanahan's method. (Bottom panel) Bar chart representing the three strains that responded best to CaCl_2_ method. * denotes that *P*<0.05; ** denotes that *P*<0.001 for the *t* tests comparing the means. Refer to Supplementary Table S2 at http://www.bioscirep.org/bsr/033/bsr033e086add.htm for detailed statistical analysis. The *t* test showed that there was no significant difference between the use of SOB or LB media for DH5α, SCS110, TOP10 and BL21 strains despite higher average TrE for SOB media.

We next examined the effect of LB [14] and SOB [10] as the starting growth media prior to competency induction. Both media are highly similar in composition, with the exception that SOB and its variant SOC (super optimal broth with catabolite repression) additionally contained MgCl_2_, MgSO_4_, KCl and glucose. Figure 2 shows that using SOB or LB for both growing and the final plated media yielded similar TrE for all the strains studied (*P*>0.05, Figure 2), with the exception of JM109 and XL1-Blue. XL1-Blue was the only strain that responded significantly better to SOB (*P*<0.05, Figure 2, top panel). Interestingly, JM109 achieved better TrE when grown in LB than in SOB [*t* (12)=3.130, *P* = 0.009, Figure 2 top panel; the Hanahan method is normally associated with SOB]. This suggests that the additional chemicals in Hanahan's method affected the chemical induction methods for particular strains depending on their genotypes. Although Mg^2+^ ions have been demonstrated to be associated with improved competency [10], K^+^ ions present in SOB may also have a contributory effect.

### Comparison of 45 and 90 s heat-shock protocols

**Figure 3:**
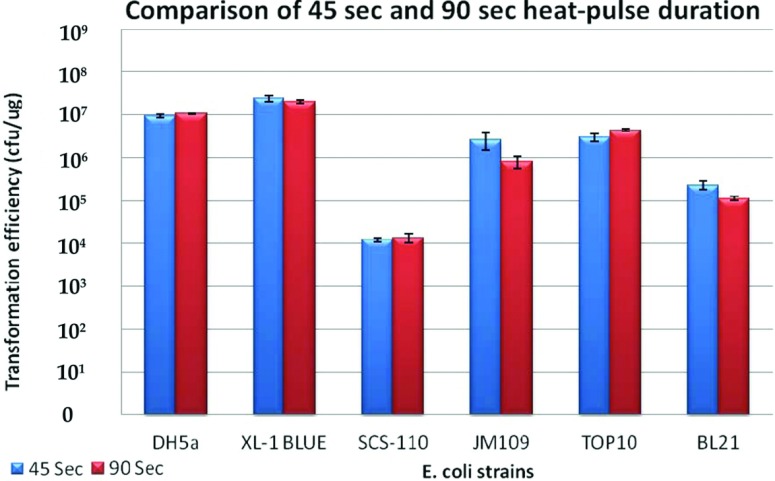
Comparison of the 45 and 90 s heat shock Bar chart showing the means, standard error and *t* test results of 45 and 90 s incubation times performed across all strains of *E. coli* made using the optimized method with SOC. The absence of significance differences indicate that the 45 or 90 s heat-pulse yielded similar transformation efficiencies across all strains tested.

The 45 s (Stratagene's recommended timing for their bacteria) was next compared with the 90 s heat-shock method [10]. We did not find significant differences between the two incubation timings for all the strains (*P*>0.05, Figure 3), suggesting that 45 s incubations were sufficient for effective pUC18 transformation for all the six strains tested.

### Boosting TrE levels through reduction of final resuspension buffer

**Figure 4:**
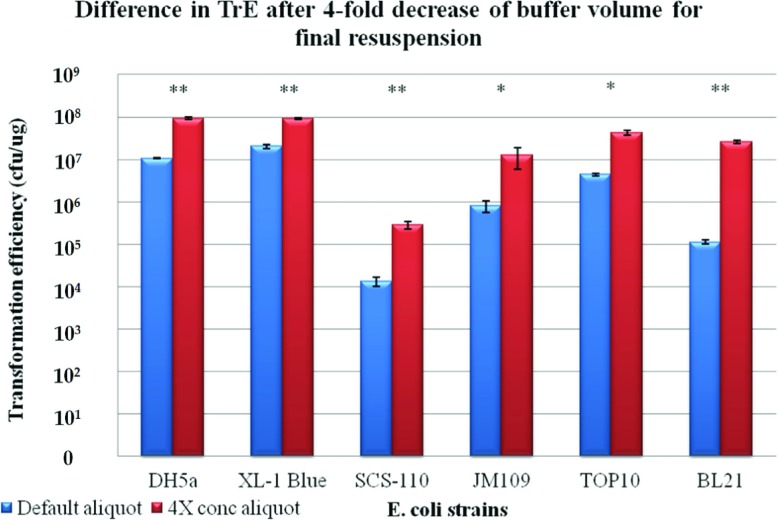
Comparison of four-fold concentration with the default final resuspension method in the optimized methods across the six *E. coli* strains Bar chart showing the means, standard errors and *t* test results of the TrE obtained from optimally induced competent bacteria that were concentrated four-fold with that of default aliquots.* denotes that *P*<0.05; ** denotes that *P*<0.001 for the *t* tests comparing the means.

Having established the optimum conditions (including growth media and heat-shock durations) for the six strains, we attempted to increase TrE by increasing the density of bacteria in each aliquot. Our very first attempt, while following Hanahan's protocol exactly ([10], using 200 μl aliquots for transformation), involved a four-fold reduction in volume of the final resuspension buffer (Figure 4). While not linear with concentration, we did obtain the minimum of a four-fold increase across all methods and strains tested (calculated in 100 μl aliquot volumes for comparisons in Figure 4). To ensure comparability, all transformations were performed using pUC18 plasmid, and ANOVA tests were carried out to ensure reproducibility (Supplementary Table S4 at at http://www.bioscirep.org/bsr/033/bsr033e086add.htm).

### Issues with respect to the current formulae for TrE

One of the issues that make comparisons across methods difficult is the fact that there is no provision for inclusion of bacteria aliquot volumes used for transformations in the estimations of TrE (cfu/μg of DNA). This may affect the estimates since the actual bacterial numbers, as measured using OD*_λ_* for log-phase determination, may vary. In addition, variations in bacterial numbers will also likely arise from the differences in final resuspension volumes. Thus, our study has demonstrated that increased bacterial density in the fixed volume aliquots can significantly boost the average TrE (Figure 4).

In summary, we propose that comparisons of TrE values across methods and strains need to ensure standardization of the following: (1) the aliquot volume used for transformation i.e. standardized to a fixed volume (e.g. 100 μl); (2) the resuspension volumes of the final freezing buffer of the various protocols taking into account the initial culture volume; (3) the use of the same plasmid (e.g. pUC 18); and (4) the growth media used.

### Conclusion

From our study comparing four commonly used chemical induction methods for producing competent bacteria, we have determined the optimal set of parameters for the most effective transformation strategy. We have obtained yields of competent bacteria that are of high cloning TrE (see Table 1 for a summary of recommended parameters and methods for each of the strains). We have also demonstrated the need for the standardization of multiple factors when comparing methods including resuspension volume ratios, transformation aliquot volumes, plasmids and growth media.

**Table 1:** Summary Table of the optimized methods and parameter for chemical induction of competency of the six *E. coli* strains Table summarizing the various parameters for chemical induction of competency used.

*E. coli* strain	Best chemical competency induction method	Growth media	*t*	*df*	*P* values (1-tailed)	Heat-shock duration	*t*	*df*	*P* values (2-tailed)	Average TrE obtained after optimization
**DH5α**	Hanahan's method	No significant difference	0.038	10	0.485	No significant difference	1.258	6	0.255	9.31×10^7^
**XL-1 Blue**	Hanahan's method	SOB > LB	4.280	16	<0.001	No significant difference	0.800	6	0.454	9.23×10^7^
**SCS110**	CaCl_2_ method	No significant difference	1.330	6	0.116	No significant difference	0.243	6	0.816	3.03×10^5^
**JM109**	Hanahan's method	LB > SOB	7.913	10	<0.001	No significant difference	1.622	6	0.156	2.26×10^7^
**TOP10**	CaCl_2_ method	No significant difference	1.156	14	0.133	No significant difference	1.811	6	0.120	4.34×10^7^
**BL21**	CaCl_2_ method	No significant difference	0.728	14	0.239	No significant difference	2.258	6	0.65	2.57×10^7^

## Online data

Supplementary data

## AUTHOR CONTRBIUTION

Weng-Tat Chan performed the majority of the laboratory work, analysis and statistical tests and drafted the paper. Chandra Verma guided the intellectual content and critical revision for the paper. David Lane provided all of the facilities required for the study, the final revision of the paper, and advice on the intellectual content. Samuel Gan is the direct supervisor of Weng-Tat Chan, he conceived the study, the overall directions of the study, the formatting, and directed the data collection and writing of the paper.
