# Supplementary material for: A comparison and optimization of methods and factors affecting the transformation of Escherichia coli
Source: Biosci Rep. 2013 Dec 12;33(6):e00086. doi: 10.1042/BSR20130098 (PMC3860579; doi:10.1042/BSR20130098)
Supplement: Supplementary data [file bsr033e086add.pdf]

Biosci. Rep. (2013) / **33** / art:e00086 / doi 10.1042/BSR20130098

OPEN ACCESS

Original Paper

Bioscience Reports

www.bioscirep.org

Bioscience Reports

SUPPLEMENTARY DATA

A comparison and optimization of methods and factors affecting the transformation of *Escherichia coli*

Weng-Tat CHAN*, Chandra S. VERMA*†‡, David P. LANE‡§ and Samuel Ken-En GAN*[1]

*Bioinformatics Institute, Agency for Science, Technology, and Research (A*STAR), Singapore 138671, †Department of Biological Sciences, National University of Singapore (NUS), Singapore 119077, ‡School of Biological Sciences, Nanyang Technological University (NTU), Singapore 639798, and §p53 Laboratory, Agency for Science, Technology, and Research (A*STAR), Singapore 138648

EXPERIMENTAL

Detailed methods of chemical induction of competency

Original $CaCl_2$ method (from Mandel and Higa [1])

Pelleted bacteria were resuspended with gentle pipetting in 25 ml (half the volume of the initial culture) of 0.1 M $CaCl_2$ (formulated in de-ionized water and autoclaved) (BDH, VWR International) and incubated on ice for 1 h. The bacteria suspension were pelleted at 4k rpm at 4°C for 8 min (Eppendorf, Model 5804R), and the supernatants were discarded. Following which, the pellets were resuspended gently in 4 ml (standardized volume) of 0.1 M $CaCl_2$ with 15% (v/v) glycerol solution and stored in 100 μl aliquots at −80°C.

Original $MgCl_2$–$CaCl_2$ method (from Sambrook and Russell [2])

Pelleted bacteria were resuspended with gentle pipetting in 15 ml of 0.1 M $MgCl_2$ (formulated in de-ionized water and autoclaved) (BDH, VWR International) and incubated on ice for 10 min. The bacteria were pelleted at 4k rpm at 4°C for 8 min (Eppendorf, Model 5804R), and the supernatants were discarded. Following which, the pellets were resuspended in 15 ml of 0.1 M $CaCl_2$, and incubated on ice for 30 min. After spinning down, the supernatant were discarded, and the pellets resuspended in 4 ml (standardized volume) of 0.1 M $CaCl_2$ with 20% (v/v) glycerol solution, and stored in 100 μl aliquots at −80°C.

Original DMSO method (from Chung and Miller [3])

Pelleted bacteria were gently resuspended in 10% volume of the initial culture of ice-cold TSB [LB broth at pH 6.1, 10% (w/v) PEG3350, 5% (v/v) DMSO, 10 mM $MgCl_2$ and 10 mM $MgSO_4$, filter sterilized with 0.45 μm filter] and incubated on ice for 30 min. The bacteria were stored in 100 μl aliquots at −80°C.

Original Hanahan's method (from Hanahan [4])

Pelleted bacteria were resuspended gently in 1/3 volume of initial starting culture (50 ml) of FSB [10 mM CH_3CO_2K at pH 7.5, 45 mM $MnCl_2$, 10 mM $CaCl_2$, 0.1 M KCl, 3 mM $[Co(NH_3)_6]Cl_3$, 10% (v/v) glycerol] and incubated on ice for 15 min. The bacteria were then pelleted at 4k rpm at 4°C for 8 min using Eppendorf, Model 5804R centrifuge and resuspended with 4 ml of FSB. 3.5% (v/v) of DMSO was added twice in intervals of 5 min [final concentration at 7% (v/v)] to the center of the suspension with gentle swirling. The bacteria suspensions were stored in 200 μl aliquots at −80°C.

Preparation of FSB

1 M CH_3CO_2K stock solution was prepared using Milli-Q grade water, and equilibrated to pH 7.5 using KOH and filtered using 0.22 μm pore-size filters prior to freezing for storage. A 10 mM CH_3CO_2K solution was prepared from the stock solution with 10% (v/v) of glycerol added. The rest of the chemicals were added into the 10 mM CH_3CO_2K solution, and the pH was adjusted to 6.4 using 0.1 N of HCL. Extreme care was taken to ensure that the pH values did not fall below 6.4 or a new buffer using new reagents was made. The pH of the buffer was allowed to drift from 6.4 for a ~1–2 days before finally settling at 6.1–6.2. The buffer was sterilized using a 0.22 μm pore-size filter and stored at 4°C in the dark. Care was taken to ensure that the glassware used for competent bacteria production were autoclaved and clean, and that the chemicals used were uncontaminated and recently purchased.

Detailed transformation protocols

DMSO method

100 μl of thawed DMSO competent cells were transferred to cold 14 ml round bottomed tubes (BD, Product no. 352059) and incubated with pUC18 DNA (Agilent, 200231-42) on ice for 30 min. The cell suspensions were then allowed to grow in 0.9 ml of

[1] To whom any correspondence should be addressed (email samuelg@bii.a-star.edu.sg).

© 2013 The Author(s) This is an Open Access article distributed under the terms of the Creative Commons Attribution Licence (CC-BY) (http://creativecommons.org/licenses/by/3.0/) which permits unrestricted use, distribution and reproduction in any medium, provided the original work is properly cited.

Table S1 OD readings of the batches of competent cells produced for comparison of the four methods
Table showing the various OD_{600} readings obtained from the initial starting culture grown for each batch of bacteria produced using the respective methods. The number of different batches produced using the different methods are represented by *n*.

Method	Strain	*n*	Batch 1 OD_{600} readings	Batch 2 OD_{600} readings	Batch 3 OD_{600} readings	Batch 4 OD_{600} readings
**$MgCl_2$–$CaCl_2$**	DH5α	4	0.404	0.297	0.337	0.337
	XL-1 Blue	4	0.364	0.357	0.32	0.32
	SCS110	4	0.301	0.367	0.301	0.301
	JM109	4	0.444	0.444	0.481	0.481
	TOP10	4	0.387	0.387	0.3	0.3
	BL21	4	0.443	0.443	0.358	0.358
**DMSO**	DH5α	4	0.337	0.306	0.337	0.337
	XL-1 Blue	4	0.32	0.356	0.32	0.32
	SCS110	4	0.301	0.355	0.301	0.031
	JM109	4	0.444	0.444	0.481	0.481
	TOP10	4	0.387	0.387	0.3	0.3
	BL21	4	0.443	0.443	0.412	0.412
**Hanahan's (standardized)**	DH5α	4	0.327	0.31	0.396	0.337
	XL-1 Blue	4	0.318	0.31	0.324	0.32
	SCS110	4	0.301	0.355	0.301	0.301
	JM109	4	0.444	0.444	0.481	0.481
	TOP10	4	0.387	0.387	0.3	0.3
	BL21	4	0.443	0.443	0.412	0.412
**$CaCl_2$**	DH5α	4	0.363	0.363	0.363	0.363
	XL-1 Blue	4	0.367	0.367	0.367	0.367
	SCS110	4	0.322	0.322	0.322	0.322
	JM109	4	0.368	0.368	0.368	0.368
	TOP10	4	0.324	0.324	0.324	0.324
	BL21	4	0.357	0.357	0.357	0.357

TSB with 20 mM of glucose, at 37 °C in vigorous shaking (speed 200–220) for 1 h. The cells were then plated on LB agar plates with 100 μg/ml ampicillin (Goldbio, A-301-5) and incubated overnight at 37 °C.

Recommended 45 s heat-shock protocol by Stratagene

100 μl of competent bacteria were mixed with control pUC18 plasmid DNA (Agilent, 200231-42) in 14-ml BD Falcon Polypropylene round-bottom tubes (Falcon 2059) and incubated on ice for 30 min. A 42 °C heat shock of 45 s was applied to the tubes, followed by immediate placement on ice for 2 min. 900 μl of SOC (SOB + 20 mM glucose) media were added to the bacteria suspensions and subsequently incubated at 37 °C for 1 h. The entire suspension was plated out on LB agar plates with 100 μg/ml ampicillin (Goldbio, A-301-5) at 37 °C overnight. All transformations of commercially purchased competent bacteria utilized this protocol.

Hanahan method for transformation

200 μl of competent bacteria were mixed with control pUC18 plasmid DNA in 14-ml BD Falcon Polypropylene round-bottom tubes and incubated on ice for 30 min. A 42 °C heat shock of 90 s was applied to the bacteria, followed by immediate placement on ice for 2 min. 900 μl of SOC media were added to the bacteria suspensions and subsequently incubated at 37 °C for 1 h. The entire suspension was plated out on LB agar plates with 100 μg/ml ampicillin (Goldbio, A-301-5) using disposable spreaders at 37 °C overnight.

© 2013 The Author(s) This is an Open Access article distributed under the terms of the Creative Commons Attribution Licence (CC-BY) (http://creativecommons.org/licenses/by/3.0/) which permits unrestricted use, distribution and reproduction in any medium, provided the original work is properly cited.

Table S2 Detailed statistical analysis of the reproducibility and differences within and between the four chemical methods of induction and six strains of *E. coli*

(A) ANOVA test of the differences between the four chemical methods across the six strains of *E. coli* studied

Strain	Sum of squares	*df*	Mean square	*F*	*P* value
**DH5α**	1.514×10^{14}	3	5.046×10^{13}	6.947	0.001
**XL-1 Blue**	2.155×10^{14}	3	7.184×10^{13}	8.321	0.000
**SCS110**	8.440×10^{10}	3	2.813×10^{10}	32.303	0.000
**JM109**	3.296×10^{13}	3	1.099×10^{13}	78.713	0.000
**TOP10**	2.602×10^{14}	3	8.673×10^{13}	27.350	0.000
**BL21**	3.105×10^{11}	3	1.035×10^{11}	23.395	0.000

The tests were performed at a 95 % confidence interval. $P < 0.05$ indicates that the methods were statistically different from one another within the particular strain.

(B) Independent *t* test of the four methods performed for each particular strain

						**95 % confidence interval**	
***E. coli* strain**	**Comparison of methods**	***t***	***df***	***P* (1-tailed)**	**Mean difference**	**Lower**	**Upper**
**DH5α**	Hanahan > $MgCl_2$–$CaCl_2$	2.30	14	0.019	4.82×10^6	3.30×10^5	9.30×10^6
	Hanahan > DMSO	2.62	15	0.010	5.15×10^6	9.68×10^5	9.34×10^6
	Hanahan > $CaCl_2$	2.92	19	0.004	4.73×10^6	1.34×10^6	8.12×10^6
	**$CaCl_2$ > $MgCl_2$–$CaCl_2$**	**0.61**	**19**	**0.276**	**8.79×10^4**	**-2.15×10^5**	**3.91×10^5**
	$CaCl_2$ > DMSO	3.14	20	0.003	4.24×10^5	1.42×10^5	7.07×10^5
	$MgCl_2$–$CaCl_2$ > DMSO	12.94	15	<0.001	3.37×10^5	2.81×10^5	3.92×10^5
**XL1-Blue**	Hanahan > $MgCl_2$–$CaCl_2$	3.01	22	0.003	4.95×10^6	1.54×10^6	8.37×10^6
	Hanahan > DMSO	3.13	21	0.003	5.39×10^6	1.81×10^6	8.97×10^6
	Hanahan > $CaCl_2$	2.45	22	0.011	4.11×10^6	6.30×10^5	7.58×10^6
	$CaCl_2$ > $MgCl_2$–$CaCl_2$	2.59	22	0.008	8.46×10^5	1.69×10^5	1.52×10^6
	$CaCl_2$ > DMSO	3.83	21	<0.001	1.28×10^6	5.85×10^5	1.98×10^6
	$MgCl_2$–$CaCl_2$ > DMSO	6.27	21	<0.001	4.35×10^5	2.91×10^5	5.79×10^5
**SCS110**	$CaCl_2$ > $MgCl_2$–$CaCl_2$	5.90	15	<0.001	1.17×10^5	7.47×10^4	1.59×10^5
	$CaCl_2$ > DMSO	5.99	15	<0.001	1.19×10^5	7.64×10^4	1.61×10^5
	$CaCl_2$ > Hanahan	5.14	15	<0.001	1.03×10^5	6.00×10^4	1.45×10^5
	Hanahan > $MgCl_2$–$CaCl_2$	6.14	14	<0.001	1.44×10^4	9.35×10^3	1.94×10^4
	Hanahan > DMSO	6.98	14	<0.001	1.61×10^4	1.12×10^4	2.11×10^4
	$MgCl_2$–$CaCl_2$> DMSO	4.63	14	<0.001	1.75×10^3	9.39×10^2	2.56×10^3
**JM109**	Hanahan > $MgCl_2$–$CaCl_2$	11.24	15	<0.001	2.37×10^6	1.92×10^6	2.82×10^6
	Hanahan > DMSO	12.47	16	<0.001	2.46×10^6	2.04×10^6	2.87×10^6
	Hanahan > $CaCl_2$	6.23	15	<0.001	1.63×10^6	1.07×10^6	2.19×10^6
	$CaCl_2$ > $MgCl_2$–$CaCl_2$	4.83	16	<0.001	7.44×10^5	4.18×10^5	1.07×10^6
	$CaCl_2$ > DMSO	5.81	17	<0.001	8.28×10^5	5.28×10^5	1.13×10^6
	$MgCl_2$–$CaCl_2$ > DMSO	2.15	17	0.023	8.41×10^4	1.55×10^3	1.67×10^5
**TOP 10**	$CaCl_2$ > $MgCl_2$–$CaCl_2$	5.18	24	<0.001	5.14×10^6	3.09×10^6	7.18×10^6
	$CaCl_2$ > DMSO	4.59	20	<0.001	5.40×10^6	2.94×10^6	7.85×10^6
	$CaCl_2$ > Hanahan	5.85	26	<0.001	5.39×10^6	3.50×10^6	7.28×10^6
	$MgCl_2$–$CaCl_2$ > DMSO	2.89	22	0.004	2.61×10^5	7.37×10^4	4.49×10^5
	$MgCl_2$–$CaCl_2$ > Hanahan	3.56	28	0.001	2.53×10^5	1.07×10^5	3.98×10^5
	Hanahan > DMSO	2.00	24	0.029	8.75×10^3	-2.96×10^2	1.78×10^4
**BL21**	$CaCl_2$ > $MgCl_2$–$CaCl_2$	6.68	19	<0.001	2.11×10^5	1.45×10^5	2.77×10^5
	$CaCl_2$ > DMSO	5.75	17	<0.001	2.11×10^5	1.33×10^5	2.88×10^5
	$CaCl_2$ > Hanahan	2.20	23	0.038	7.24×10^4	4.41×10^3	1.40×10^5
	Hanahan > $MgCl_2$–$CaCl_2$	5.20	18	<0.001	1.38×10^5	8.24×10^4	1.94×10^5
	Hanahan > DMSO	4.48	16	<0.001	1.38×10^5	7.28×10^4	2.04×10^5
	$MgCl_2$–$CaCl_2$> DMSO	NA	–	–	–	–	–

The tests were performed at 95 % confidence. Absence of a *P* value indicates the absence of transformants. $P < 0.05$ showed that the strains responded differently to the various methods, with the exception of DH5α, which responded similarly to both $CaCl_2$ and $MgCl_2$–$CaCl_2$ methods (bold).

© 2013 The Author(s) This is an Open Access article distributed under the terms of the Creative Commons Attribution Licence (CC-BY) (http://creativecommons.org/licenses/by/3.0/) which permits unrestricted use, distribution and reproduction in any medium, provided the original work is properly cited.

Table S2 Continue

(C) ANOVA test of reproducibility for the four methods for the six *E. coli* strains

Methods	*E. coli* strains	Sum of squares	*df*	Mean square	*F*	*P values*
**$MgCl_2$–$CaCl_2$ method**	DH5α	1.593×10^{10}	3	5.312×10^{9}	0.999	0.479
	XL1-Blue	1.371×10^{11}	3	4.569×10^{10}	0.832	0.513
	SCS110	4750000.000	3	1583333.333	1.949	0.264
	JM109	1.779×10^{10}	3	5.931×10^{9}	0.370	0.779
	TOP10	2.232×10^{11}	3	7.439×10^{10}	0.895	0.477
	BL21	0.000	3	0.000	.	.
**DMSO method**	**DH5α**	**5.337×10^{9}**	**3**	**1.779×10^{9}**	**19.331**	**0.004**
	XL1-Blue	5.879×10^{8}	3	1.960×10^{8}	1.075	0.419
	SCS110	0.000	3	0.000	.	.
	JM109	4.903×10^{8}	3	1.634×10^{8}	0.039	0.989
	TOP10	1.055×10^{7}	3	3515625.000	0.783	0.546
	BL21	0.000	3	0.000	.	.
**Hanahan's method**	DH5α	5.040×10^{11}	3	1.680×10^{11}	1.522	0.338
	XL1-Blue	8.225×10^{13}	3	2.742×10^{13}	0.798	0.529
	SCS110	1.174×10^{8}	3	3.913×10^{7}	0.862	0.530
	JM109	1.487×10^{12}	3	4.956×10^{11}	1.588	0.325
	TOP10	4.000×10^{7}	3	1.333×10^{7}	1.600	0.285
	BL21	1.577×10^{10}	3	5.256×10^{9}	0.926	0.471
**$CaCl_2$ method**	DH5α	1.778×10^{11}	3	5.926×10^{10}	0.333	0.802
	XL1-Blue	3.090×10^{12}	3	1.030×10^{12}	0.792	0.532
	SCS110	1.452×10^{10}	3	4.840×10^{9}	2.321	0.192
	JM109	3.008×10^{11}	3	1.003×10^{11}	0.383	0.770
	TOP10	4.669×10^{13}	3	1.556×10^{13}	1.192	0.373
	BL21	2.581×10^{10}	3	8.603×10^{9}	1.876	0.204

The tests were performed at a 95% confidence level. $P > 0.05$ indicates that the various batches of bacteria were not different from one another (i.e. reproducible within the same method). Absence of *P* values indicates that there were no transformants observed. The bolded regions shows where $P < 0.05$ (i.e. the batches are not reproducible within the same method).

© 2013 The Author(s) This is an Open Access article distributed under the terms of the Creative Commons Attribution Licence (CC-BY) (http://creativecommons.org/licenses/by/3.0/) which permits unrestricted use, distribution and reproduction in any medium, provided the original work is properly cited.

Table S3 Summary of the genotypes for the six *E. coli* strains

Genotypes were retrieved from: DH5α – Invitrogen, Cat # 12297-016, XL-1 Blue – Stratagene, Cat # 200247, SCS110 – Stratagene, Cat # 200249, JM109 – Promega, Cat # L2001, TOP10 – Invitrogen, Cat # C4040-10, and BL21 – Invitrogen, Cat # C6060-03. Common modified genes were grouped accordingly to the methods that the strains responded best to.

	**Hanahan's method**			**$CaCl_2$ method**		
***E. coli* strains**	**DH5α**	**XL-1 Blue**	**JM109**	**TOP10**	**SCS110**	**BL21**
Genotype modifications exclusive to *E. coli* strains sensitive to the method of production	*relA1*	*relA1*	*relA1*	*gal*U/*gal*K	*gal*K/*gal*T	*gal*
	gyrA96	*gyrA96*	*gyrA96*			
Genotype modification shared across the multiple strain of *E. coli studied*	*F-*		*[F' tra*D36, *pro*AB, *laq*IqZΔM15]	*F-*	*[F' traD36 proAB lacI*^{q}ZΔM15]	*F-*
	recA1	*recA1*	*recA1*	*recA1*		
	endA1	*endA1*	*endA1*	*endA1*	*endA1*	
	*hsd*R17 (rk-, mk+)	*hsdR17*	*hsd*R17 (rk-, mk+)	*Δ(mrr-hsd*RMS-*mcr*BC)		*hsd*SB(rB-, mB-)
	Δ(lacZ)M15	*lac*	*Δ*(*lac-pro*AB)	*Δlac*X74	*lacY*	
	thi-1	*thi-1*	*thi-1*		*thi-1*	
				(StrR)		*(StrR)*
		*sup*E44	*sup*E44		*supE44D*	
					dcm	*dcm*
	Φ80		*Φ80lac*ZΔM15			
Genotype modifications exclusive to individual strains	fhuA2			mcrA	rpsL	ompT
	Δ*(argF-lacZ)*			araD139	thr	(DE3)
	U169			Δ(*ara leu*)	leu	pLysS
	phoA			7697	ara	(CamR)
	glnV44			rpsL	tonA	
				nupG	tsx	
					dam	
					(lac-proAB)	

Table S4 ANOVA test for reproducibility of optimized competent bacteria

Table showing ANOVA of the different batches of bacteria produced using the optimized protocol with 4-fold concentration. Test was performed at 95% confidence interval. $P > 0.05$ indicates that the batches were reproducible.

Optimized method for each strain	Sum of squares	*df*	Mean square	*F*	*P values*
**DH5α – Hanahan**	2.632×10^{13}	1	2.632×10^{13}	0.160	0.728
**XL-1 Blue – Hanahan**	9.610×10^{12}	1	9.610×10^{12}	0.153	0.733
**SCS110 – $CaCl_2$**	2.756×1^{9}	1	2.756×10^{9}	0.139	0.745
**JM109 – Hanahan LB**	1.232×10^{12}	1	1.232×10^{12}	0.006	0.945
**TOP10 – $CaCl_2$**	5.595×10^{13}	1	5.595×10^{13}	0.168	0.722
**BL21 – $CaCl_2$**	1.722×10^{13}	1	1.722×10^{13}	0.552	0.535

© 2013 The Author(s) This is an Open Access article distributed under the terms of the Creative Commons Attribution Licence (CC-BY) (http://creativecommons.org/licenses/by/3.0/) which permits unrestricted use, distribution and reproduction in any medium, provided the original work is properly cited.

REFERENCES

1 Mandel, M. and Higa, A. (1970) Calcium-dependent bacteriophage DNA infection. J. Mol. Biol. **53**, 159–162

2 Sambrook, J. and Russell, D. W. (2001) Molecular Cloning: a Laboratory Manual, Cold Spring Harbor Laboratory Press, Volume 1

3 Chung, C. T. and Miller, R. H. (1988) A rapid and convenient method for the preparation and storage of competent bacterial cells. Nucleic Acids Res. **16**, 3580

4 Hanahan, D. (1983) Studies on transformation of *Escherichia coli* with plasmids. J. Mol. Biol. **166**, 557–580

Received 27 September 2013/13 November 2013; accepted 14 November 2013

Published as Immediate Publication 14 November 2013, doi 10.1042/BSR20130098

© 2013 The Author(s) This is an Open Access article distributed under the terms of the Creative Commons Attribution Licence (CC-BY) (http://creativecommons.org/licenses/by/3.0/) which permits unrestricted use, distribution and reproduction in any medium, provided the original work is properly cited.
